# Pneumocystis Pneumonia With Multiple Centrilobular Pulmonary Nodules and Lack of Ground-Glass Attenuation on High-Resolution Computed Tomography

**DOI:** 10.7759/cureus.35565

**Published:** 2023-02-28

**Authors:** Ryuichiro Takaki, Kosaku Komiya, Nobuhiro Fujishima, Marimu Yamanaka, Atsushi Yokoyama, Kazufumi Hiramatsu, Jun-ichi Kadota

**Affiliations:** 1 Respiratory Medicine and Infectious Diseases, Oita University, Yufu, JPN; 2 Respiratory Medicine and Infectious Diseases, Oita University Faculty of Medicine, Yufu, JPN; 3 Research Center for Global and Local Infectious Diseases, Oita University Faculty of Medicine, Yufu, JPN

**Keywords:** hrct, aids, hiv, ground-glass attenuation, pneumocystis jirovecii pneumonia

## Abstract

*Pneumocystis jirovecii *pneumonia (PCP) typically presents with diffuse ground-glass attenuation (GGA) in both lungs on high-resolution CT (HRCT). While other radiological features, including cysts and air-space consolidation, may be found, the absence of GGA has a high negative predictive value for PCP in patients with AIDS. We report a case of PCP in a male patient who visited our hospital with a subacute, non-productive cough. He had never been diagnosed with an HIV infection. Although his HRCT scan revealed multiple centrilobular nodules without GGA, *Pneumocystis jirovecii *was detected in the bronchoalveolar lavage (BAL), and no other additional pathogens were identified. The patient was diagnosed with PCP associated with AIDS after a high plasma HIV-RNA titer and low CD4+ cell count were confirmed. Physicians need to be aware of this atypical radiological presentation of PCP associated with AIDS.

## Introduction

*Pneumocystis jirovecii *pneumonia (PCP) is a fatal pulmonary infection and the most common complication of HIV/AIDS [1]. Since appropriate prophylaxis can prevent PCP development, patients who were already diagnosed with HIV/AIDS and are well-managed rarely suffer from this infectious disease [2]. Instead, PCP may indicate the first manifestation of AIDS.

Patients with PCP generally complain of low-grade fever, subacute dyspnea, and a non-productive or minimally productive cough. High-resolution CT (HRCT) typically shows diffuse ground-glass attenuation (GGA) in both lungs, which is thought to reflect the accumulation of cellular debris, fibrin, and *Pneumocystis jirovecii *within the alveolar spaces [3]. While other radiological features, including pulmonary cysts and consolidation, may be present, diffuse GGA has a high sensitivity for PCP in patients with AIDS. While no large-scale studies have described the radiological features of PCP, a case series study has demonstrated that the presence of GGA on HRCT indicates a 100% sensitivity for PCP [4]; if this feature is not observed, it might lead to the elimination of PCP from the differential diagnoses.

We report a case of a patient with PCP who presented with multiple centrilobular nodules without GGA and was concurrently diagnosed with AIDS. The radiological features in immunocompromised hosts vary widely, and we also engage in a discussion of the mechanism of this unusual radiological presentation.

## Case presentation

A male in his 50s visited our hospital with a one-month history of persistent non-productive cough. He had a history of hypertension and had been in a homosexual relationship with a young man for the past year, but had never been diagnosed with HIV infection. The physical examination revealed a body temperature of 36.7 °C, a percutaneous oxygen saturation (SpO_2_) of 94% without supplemental oxygenation, a blood pressure of 133/88 mmHg, and a heart rate of 84 beats/minute. The arterial blood gas analysis performed without oxygen supplementation revealed that the patient was in a mild hypoxic state (pH: 7.490; PaO_2_: 58 Torr; PaCO_2_: 39 Torr; HCO_3_: 30 mmol/L). The laboratory blood tests showed high levels of C-reactive protein (2.80 mg/dL), and normal levels of white blood cell count (4,140/μL), hemoglobin (13.6 g/dL), blood urea nitrogen (12.5 mg/dL), and serum creatinine (0.88 mg/dL). The test results for *Candida albicans* antigen, *Cryptococcus *antigen, interferon-γ release assay (T-SPOT.TB test, Oxford Immunotec, Oxford, UK) to screen for tuberculosis infection, anti-MAC antibody, and cytomegalovirus antigenemia test (C7-HRP) were negative, and elevated levels of beta-D glucan (421 pg/mL) and KL-6 (2,173 U/mL) were noted.

**Figure 1:**
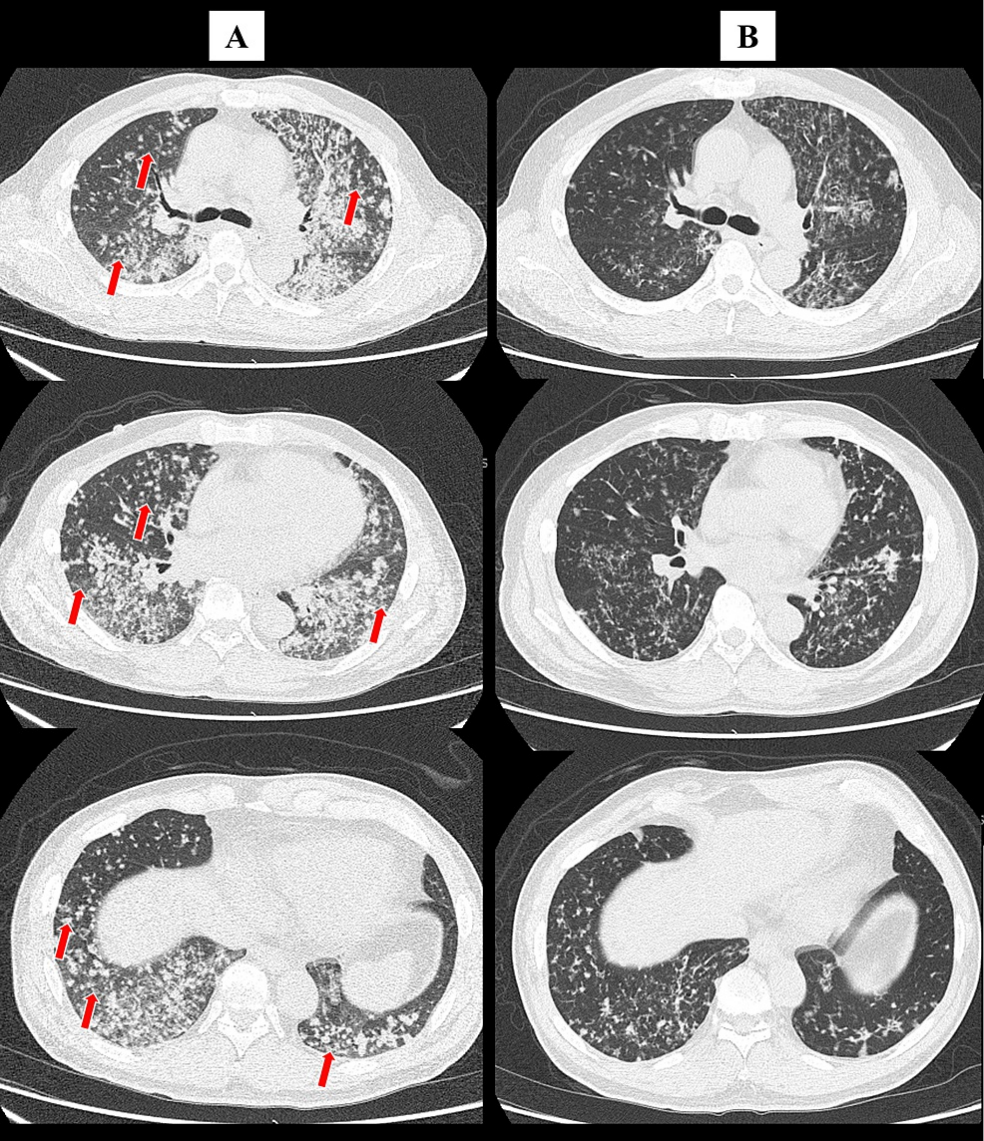
Chest high-resolution CT showing the upper, middle, and lower lung lobes upon the patient’s first visit to our hospital (A), and five days after treatment with trimethoprim-sulfamethoxazole (B) The arrows on each figure indicate centrilobular nodules CT: computed tomography

**Figure 2:**
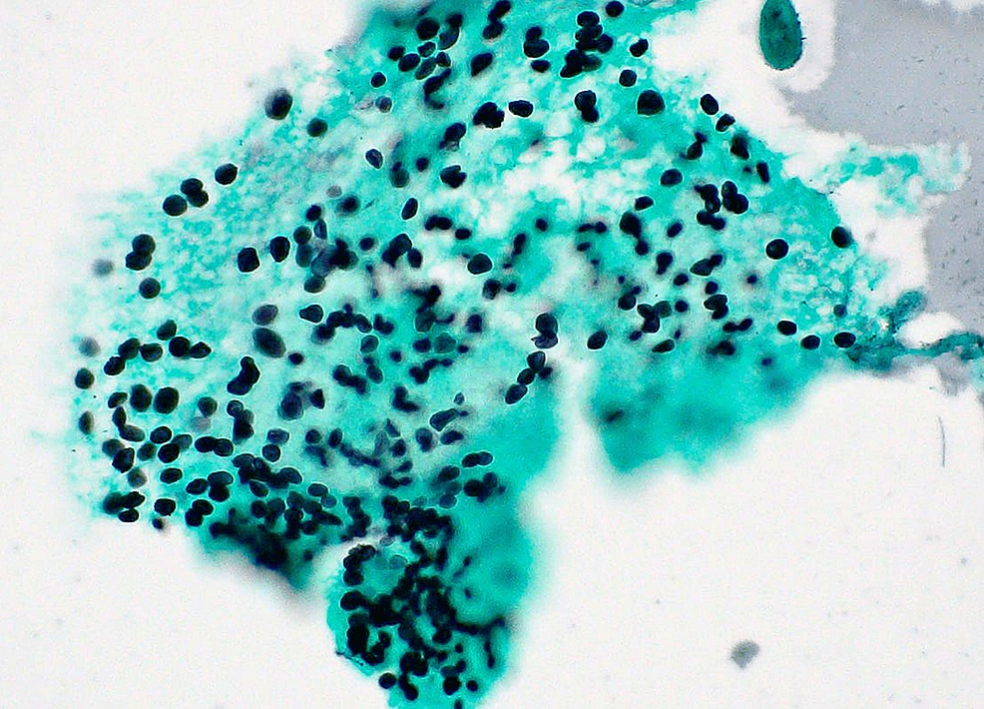
Grocott staining demonstrates capsular dots in bronchoalveolar lavage fluid, consistent with Pneumocystis jirovecii

A chest HRCT revealed multiple centrilobular nodules in both lungs without GGA (Figure 1A). Based on the presence of characteristic HRCT features, including a “tree-in-bud appearance” suggestive of centrilobular nodules, we initially suspected mycobacterium lung infection; however, the results of the sputum culture and PCR for acid-fast bacilli were both negative. Bronchoscopy with bronchoalveolar lavage (BAL) detected *Pneumocystis jirovecii* with Grocott staining (Figure 2). No other pathogens were found on the BAL culture. An HIV screening test performed 23 days before admission was positive, and a high HIV-RNA titer (3.2 × 105 copies/mL) and low CD4+ cell count (70 cells/μL) were confirmed.

We diagnosed the patient with PCP associated with AIDS and immediately started treatment with trimethoprim-sulfamethoxazole. Oral prednisolone was concurrently administered as his PaO_2_ was <70 mmHg [5]. The therapy with trimethoprim-sulfamethoxazole was continued for 21 days. HRCT was performed five days after treatment to radiologically confirm treatment efficacy, and the outcomes showed that the multiple centrilobular nodules had resolved (Figure 1B). He is now being treated with bictegravir/emtricitabine/tenofovir/alafenamide for HIV infection.

## Discussion

This patient with PCP presented with multiple centrilobular pulmonary nodules without GGA on HRCT. Nevertheless, *Pneumocystis jirovecii* was confirmed with BAL analysis, and no other organisms, including acid-fast bacilli or fungi, were detected. Furthermore, antibiotics other than anti-*Pneumocystis jirovecii *agents had never been administered to this patient. Consequently, a diagnosis of pneumonia caused solely by *Pneumocystis jirovecii* was made.

The radiological features of infectious diseases in the lungs may vary depending on the host's immune competence. For example, immunocompromised patients with pulmonary tuberculosis are likely to exhibit a more atypical pulmonary involvement, including fewer areas of cavities or calcification and a higher prevalence of lymphadenopathy than immunocompetent patients [6,7]. In *Mycoplasma pneumoniae (M. pneumoniae)* pneumonia, it has been reported that the positivity of the purified protein derivative test in patients with a nodular pattern on chest CT was higher than that in patients with pulmonary consolidation or a GGA pattern on CT [8]. This suggests that the radiological features of *M. pneumoniae* pneumonia may be altered by the level of host cell-mediated immunity and the predominance of Th1 or Th2 inflammation [9] and that nodules reflecting granulomatous lesions are less likely to be observed in hosts with deteriorated cell-mediated immunity.

One study has demonstrated an association between chest CT features and CD4+ cell count in 50 patients with AIDS in a retrospective fashion [10]. The results showed that the CD4+ cell count (median: 25, range: 2-273 cells/μL) in patients with a GGA-dominated pattern was significantly higher than in those with a pulmonary cyst-dominated pattern (median: 6, range: 2-36 cells/μL). Other studies have indicated that a low CD4 count or CD4/CD8 ratio in the peripheral blood is associated with an emphysematous lesion on chest CT [11,12]. Based on the results from these observational studies, a low CD4+ cell count may be related to cystic or emphysematous appearances, whereas the pathological mechanism remains unclear. In contrast, the relationship between CD4+ cell count and GGA on HRCT still lacks evidence.

The patient in the current study had a low CD4+ cell count (70 cells/μL); however, it seems to be a relatively high count for patients with PCP and AIDS and falls within the range of values exhibited by the GGA-dominated pattern group shown in the former study [10]. There is only one published case report of PCP with similar centrilobular nodules without GGA on chest CT [13]. This patient’s CD4+ cell count was 15 cells/μL; this value cannot be conclusively classified into the range of values in the GGA-dominated pattern group or the lung cyst-dominated pattern group [10]. Presumably, the radiological manifestations cannot be predominantly explained by the CD4+ cell count; the lung’s local immune responses including the predominance of Th1 or Th2 inflammation and viral load need to be analyzed to clarify the associations.

## Conclusions

Although the absence of GGA on HRCT has a high negative predictive value for PCP in patients with HIV/AIDS, the value would not be 100%; thus, physicians need to be aware that PCP cannot be eliminated from the differential diagnoses based on radiographic features in these patients. Further research is required to determine the factors associated with the chest CT features of PCP.
